# Telomere Attrition in Induced Pluripotent Stem Cell-Derived Neurons From ALS/FTD-Related *C9ORF72* Repeat Expansion Carriers

**DOI:** 10.3389/fcell.2022.874323

**Published:** 2022-06-13

**Authors:** Hayley Robinson, Sk Imran Ali, Martha Elena Diaz-Hernandez, Rodrigo Lopez-Gonzalez

**Affiliations:** ^1^ Department of Neurosciences, Lerner Research Institute, Cleveland Clinic, Cleveland, OH, United States; ^2^ Department of Orthopaedics, Emory University, Atlanta, GA, United States; ^3^ Atlanta VA Medical Center, Decatur, GA, United States

**Keywords:** induced pluripotent stem cells, motor neuron differentiation, telomeres, C9orf72, Amyotrophic lateral sclerosis, Frontotemporal dementia

## Abstract

The GGGGCC (G4C2) repeat expansion in *C9ORF72* is the most common genetic cause of amyotrophic lateral sclerosis (ALS) and frontotemporal dementia (FTD). Dysregulated DNA damage response and the generation of reactive oxygen species (ROS) have been postulated as major drivers of toxicity in *C9ORF72* pathogenesis. Telomeres are tandem-repeated nucleotide sequences that are located at the end of chromosomes and protect them from degradation. Interestingly, it has been established that telomeres are sensitive to ROS. Here, we analyzed telomere length in neurons and neural progenitor cells from several induced pluripotent stem cell (iPSC) lines from control subjects and *C9ORF72* repeat expansion carriers. We found an age-dependent decrease in telomere length in two-month-old iPSC-derived motor neurons from *C9ORF72* carriers as compared to control subjects and a dysregulation in the protein levels of shelterin complex members TRF2 and POT1.

## Introduction

Amyotrophic lateral sclerosis (ALS) and Frontotemporal dementia (FTD) are two neurodegenerative diseases that share common genetic and pathogenic features. The GGGGCC (G4C2) repeat expansion in the chromosome 9 open reading frame 72 (*C9ORF72*) is the most common familial form of ALS and FTD (DeJesus-Hernandez et al., 2011; Renton et al., 2011). As we began to explore the mechanisms that drive disease pathogenesis in *C9ORF72* repeat expansion, we found that DNA damage and the DNA damage response (DDR) have a central role in disease pathogenesis. Increases in DNA damage and dysregulation of DNA repair pathways have been reported in several *in vitro* and *in vivo* models of *C9ORF72*-related ALS/FTD (Lopez-Gonzalez et al., 2016; Farg et al., 2017; Lopez-Gonzalez et al., 2019; Maor-Nof et al., 2021; Szebényi et al., 2021). Strikingly, we and others have found that reducing levels of P53, a key regulator of genome instability, can rescue neuronal death in *C9ORF72* neurons (Lopez-Gonzalez et al., 2019; Maor-Nof et al., 2021). Interestingly, reactive oxygen species (ROS) contribute to DNA damage in induced pluripotent stem cell (iPSC)-derived neurons from *C9ORF72* repeat expansion carriers (Lopez-Gonzalez et al., 2016), and there is evidence that ROS can trigger telomere attrition (Guan et al., 2015).

Telomeres are DNA-protein complexes located at the end of mammalian chromosomes. Their main function is to protect DNA from degradation. Telomere structure consists of TTAGGG repeated nucleotide sequences associated with 6 proteins that form the shelterin complex: TRF1, TRF2, POT1, RAP1, TPP1 and TIN2 (Penev et al., 2022). The shelterin complex provides protection to chromosome ends by binding to double- and single-stranded DNA to prevent the activation of the DDR. TRF1 and TFR2 bind to double-strand telomere DNA, and POT1 binds to single-strand telomere DNA (Markiewicz-Potoczny et al., 2021). Dysregulation of shelterin complex proteins can leave unprotected chromosome ends prone to telomere attrition (Miller et al., 2012).

Telomere attrition has been analyzed in other neurodegenerative conditions, including Alzheimer’s disease (AD) and Parkinson’s disease (PD). For instance, a study that compared 260 PD patients to 270 age-matched controls found shorter leucocyte telomere length in PD patients (Wu et al., 2020). Shortened telomere length has also been reported in AD patients as compared with age-matched controls (Scarabino et al., 2017). Dhillon et al. showed that ApoE4 homozygous AD patients have shorter telomeres compared to controls (Dhillon et al., 2020). Telomere analysis of patients with Huntington’s disease and FTD showed telomere attrition (Kota et al., 2015). Nonetheless, most of these analyses have been conducted in blood cells and brain sections with a mixture of different neuronal populations. To date, there are no studies that analyze telomere length in neuronal populations.

Here we analyzed telomere length and the expression levels of shelterin complex members TRF1, TRF2 and POT1, in iPSC-derived motor neurons from *C9ORF72* repeat expansion carriers and healthy controls. This analysis was performed at different stages of motor neuron (MN) differentiation from iPSCs, including neuroepithelial cells (NEP), motor neuron progenitors (MNPs), and post-mitotic MNs. We chose this model because MNs are a disease-relevant neuronal population and their differentiation protocol is well-characterized and produces highly pure MN populations. We found differential regulation in the levels of shelterin complex members and a decrease in telomere length in differentiated 2-month-old MNs, indicating that *C9ORF72* repeat expansion-induced telomere attrition can further contribute to genome instability.

## Materials and Methods

### Motor Neuron Differentiation From iPSC Lines

In this study we used 3 control iPSC lines: 37L20, 35L5 and 35L11, with ages at biopsy between 45 and 56 years-old (two males and one female), and 3 lines from *C9ORF72* carriers: 40L3, 16L14 and 42L11, with ages at biopsy between 39 and 59 years-old (two males and one female), All lines have been fully characterized and published previously (Zhang et al., 2013; Freibaum et al., 2015; Lopez-Gonzalez et al., 2016). Motor neurons were differentiated as described previously (Lopez-Gonzalez et al., 2019). Briefly, iPSCs were plated and expanded in mTSER1 medium (Stem Cell Technologies) in Matrigel-coated wells. Twenty-four hours after plating, the culture medium was replaced with neuroepithelial progenitor (NEP) medium, DMEM/F12, neurobasal medium at 1:1, 0.5X N2, 0.5X B27, 0.1 mM ascorbic acid (Sigma), 1X Glutamax (Invitrogen), 3 μM CHIR99021 (StemCell technologies), 2 μM DMH1 (Tocris Bioscience), and 2 μM SB431542 (StemCell technologies). After 6 days, NEPs were dissociated with accutase, split 1:6 into Matrigel-coated wells, and cultured for 6 days in motor neuron progenitor induction medium (NEP with 0.1 μM retinoic acid and 0.5 μM purmorphamine, both from Stem Cell technologies). Motor neuron progenitors were dissociated with accutase to generate suspension cultures. After 6 days, the cultures were dissociated into single cells, plated on laminin-coated plates/coverslips in motor neuron differentiation medium containing 0.5 μM retinoic acid, 0.1 μM purmorphamine, and 0.1 μM Compound E (Calbiochem). Post-mitotic motor neurons were cultured up to two months and analyzed at 1 month, 1.5 months, and 2 months.

### Western Blot Analysis

Human iPSCs, NEP, MNP, and MN cultures were lysed with Pierce™ RIPA Buffer (Thermo Scientific) supplemented with Halt™ Protease and Phosphatase Inhibitor Cocktail (Thermo Scientific). Protein lysates (25 µg) were analyzed by SDS-PAGE and immunoblotted to detect the expression of specific proteins using the primary antibodies listed in Table 1 and incubated overnight at room temperature. Membranes were then washed with TBS-T and incubated with appropriate anti-mouse or anti-rabbit IR-Dye secondary antibodies (LI-COR Biosciences). Membranes were imaged with an Odyssey^®^ DLx Imaging System (LI-COR Biosciences) and images were analyzed by Image Studio (LI-COR Biosciences). Equal loading was evaluated by assessing beta-actin levels.

**TABLE 1:** List of antibodies used for western blot in this study.

Antibody	Source sp.	Vendor	Catalogue No.
TRF2	Mouse	Santa Cruz Biotech	sc-52968
POT1	Rabbit	Proteintech	10581-1-AP
β-Actin	Rabbit	Abclonal	AC038

### RNA Extraction and Quantitative Real-Time PCR

Total RNA from iPSC cells and iPSC-derived motor neurons were isolated using PureLink™ RNA Mini Kits (Invitrogen) as per manufacturer’s instructions. RNA concentration and purity were measured with a NanoDrop™ One Microvolume UV-Vis Spectrophotometer (Thermo Scientific) and reverse transcribed to synthesize cDNA by SuperScript™ IV First-Strand Synthesis System (Invitrogen) according to manufacturer’s instructions using C1000 Thermal Cycler (Biorad). cDNA (10 ng) was used for quantitative real-time PCR by SYBR™ Green PCR Master Mix (Applied Biosystem) in a QuantStudio™ 6 Flex Real-Time PCR System using the primers listed in Table 2. GAPDH was used as a housekeeping gene and Ct values for each gene were normalized to that of GAPDH. Relative expression was analyzed by the 2 delta-delta Ct method.

**TABLE 2:** List of primers used in this study.

qPCR primers	Primer Sequences (5’ → 3′)
POT1 forward	TCA​GAT​GTT​ATC​TGT​CAA​TCA​GAA​CCT
POT1 reverse	TGT​TGA​CAT​CTT​TCT​ACC​TCG​TAT​AAT​GA
TRF1 forward	GCT​GTT​TGT​ATG​GAA​AAT​GGC
TRF1 reverse	CCG​CTG​CCT​TCA​TTA​GAA​AG
TRF2 forward	GAC​CTT​CCA​GCA​GAA​GAT​GCT
TRF2 reverse	GTT​GGA​GGA​TTC​CGT​AGC​TG
GAPDH forward	TGC​ACC​ACC​ACC​TGC​TTA​GC
GAPDH reverse	GGC​ATG​GAC​TGT​GGT​CAT​GAG
TEL forward	CGG​TTT​GTT​TGG​GTT​TGG​GTT​TGG​GTT​TGG​GTT​TGG​GTT
TEL reverse	GGC​TTG​CCT​TAC​CCT​TAC​CCT​TAC​CCT​TAC​CCT​TAC​CCT
36B4 forward	CAG​CAA​GTG​GGA​AGG​TGT​AAT​CC
36B4 reverse	CCC​ATT​CTA​TCA​TCA​ACG​GGT​ACA​A
hTERT forward	GCC​GAT​TGT​GAA​CAT​GGA​CTA​CG
hTERT reverse	GCT​CGT​AGT​TGA​GCA​CGC​TGA​A

### Telomere Length Quantification

We performed telomere length quantification by qPCR as previously described by (Chuenwisad et al., 2021). Briefly, we performed genomic DNA extraction from iPSC, NEP, MNP, and MN using the PureLink Genomic DNA Mini kit (Thermo Fischer Scientific). We used 15 ng of genomic DNA with the primers listed in Table 2. We considered the ratio of copy number of telomeric repeat to the number of a single-copy gene, in this case the 36B4 gene, which encodes a ribosomal protein. The telomere-to-single copy gene (T/S) ratio give us an estimate of telomere length.

### Assessment of Telomerase Activity

Telomerase activity was measured by using the telomerase activity quantification qPCR assay kit (ScienCell, CA) according to manufacturer’s instruction. Briefly, equal number of cells were lysed using cell lysis buffer, provided by manufacturer, supplemented with HaltTM Protease and Phosphatase Inhibitor Cocktail (Thermo Scientific) and *β*-mercaptoethanol. This step released the native telomerase enzyme from cells in an *ex-vivo* condition. 0.5 μL cell lysates were then incubated with appropriate amount of 5X telomerase reaction buffer, from the kit, and nuclease free water. This reaction was performed in C1000 Touch Thermal Cycler (Biorad) at 37°C for 3 h followed by an inactivation step at 85°C for 10 min. Finally, to measure the telomere production by *ex-vivo* telomerase qPCR was performed in QuantStudio™ 6 Flex Real-Time PCR System (Applied Biosystem), according to manufacturer’s instruction, using supplied 2X GoldNStart TaqGreen qPCR master mix and telomere primer set. Relative telomerase activity was calculated by 2^−ΔCt^ method as per manufacturer’s instruction.

### Immunostaining

Human iPSCs, NEP, and MNP cultures from controls and *C9ORF72* carriers were fixed in 4% paraformaldehyde for 15 min and permeabilized with 0.3% Triton X-100 for 5 min. The cells were blocked with 5% bovine serum albumin for 30 min and incubated using the primary antibodies anti Oct4 1:500 (abclonal; cat#A7920), Sox1 1:200 (R&D systems; cat#AF3369), Olig2 1:500 (R&D systems; cat#AF2418), ChAT 1:200 (Millipore; cat#AB144P) and TUJ1 1:1000 (abclonal; cat# A17913), overnight at 4°C. Cells were then incubated with secondary antibodies Alexa Fluor 488, 568, and 647 at a 1:500 dilution for 2 h at room temperature, and washed with PBS and incubated with Hoechst for 5 min.

### Confocal Microscopy

Confocal images from iPSC, NEP, MNP, and MN were acquire with a Leica DM6 upright laser-scanning confocal microscope (Leica Microsystems). Images were processed with Leica LAS AF software.

### Statistical Analyses

Statistical analyses were performed in GraphPad Prism version 9.1 (La Jolla, CA). Two-tailed t-tests with Welch’s correction were applied to analyze differences between two means. Significant differences among multiple means were analyzed by one-way ANOVA followed by Tukey’s multiple-comparison test. Null hypotheses were rejected at *p* > 0.05.

## Results

### Analysis of Telomere Length and the Expression of Telomere Maintenance Proteins During Motor Neuronal Differentiation

**FIGURE 1:**
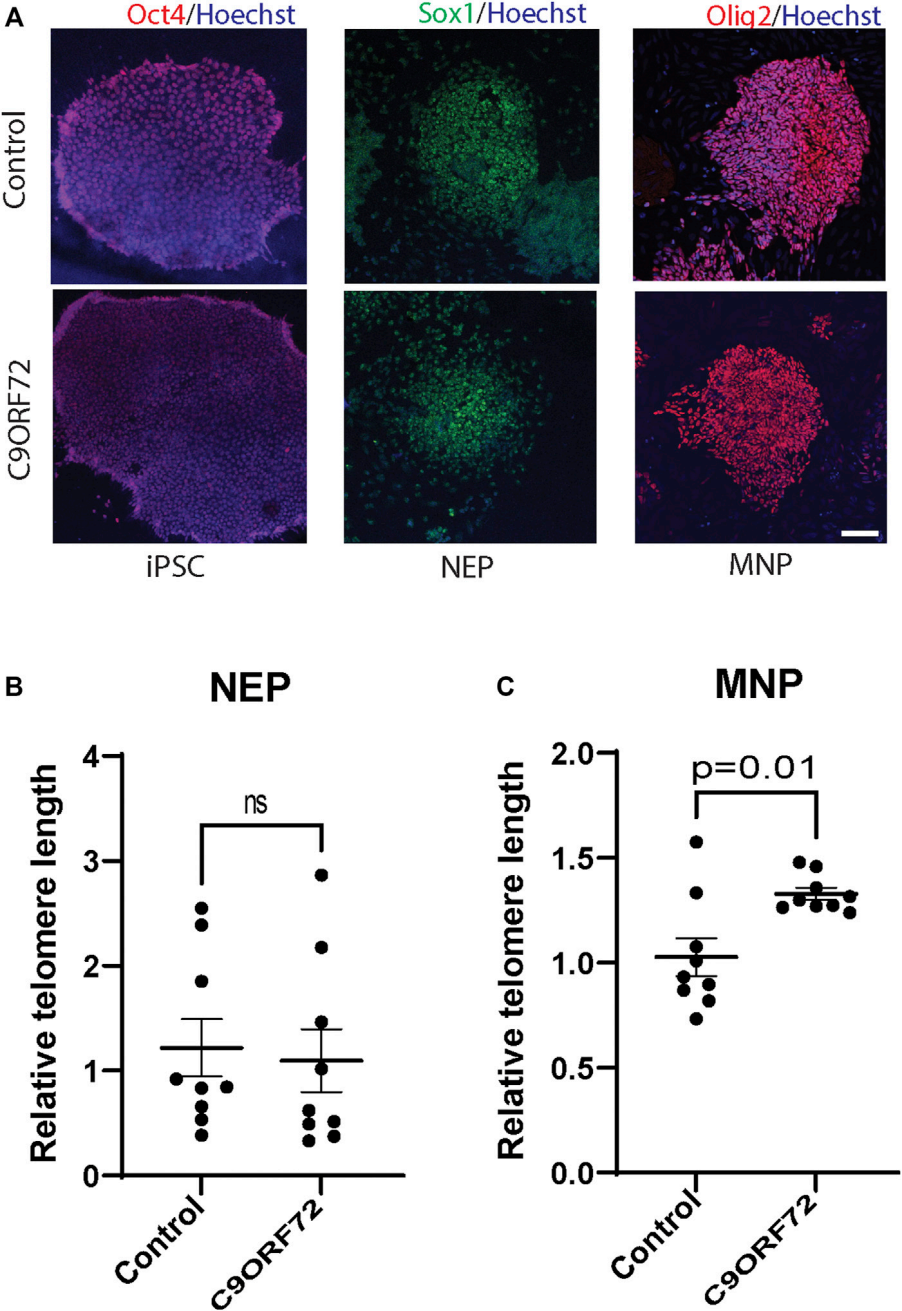
Telomere length analysis during the differentiation of iPSCs to MNs from controls and *C9ORF72* repeat expansion carriers. Representative images of control and *C9ORF72* **(A)** iPSC**s**, NEP, and MNP. Telomere length quantification in **(B)** NEP and **(C)** MNP, from 3 controls and 3 *C9ORF72* iPSC lines**,** from 3 independent differentiations. Two-tailed *t*-test with Welch’s correction was applied. ns, not significant. Scale bar = 20 µm.

**FIGURE 2:**
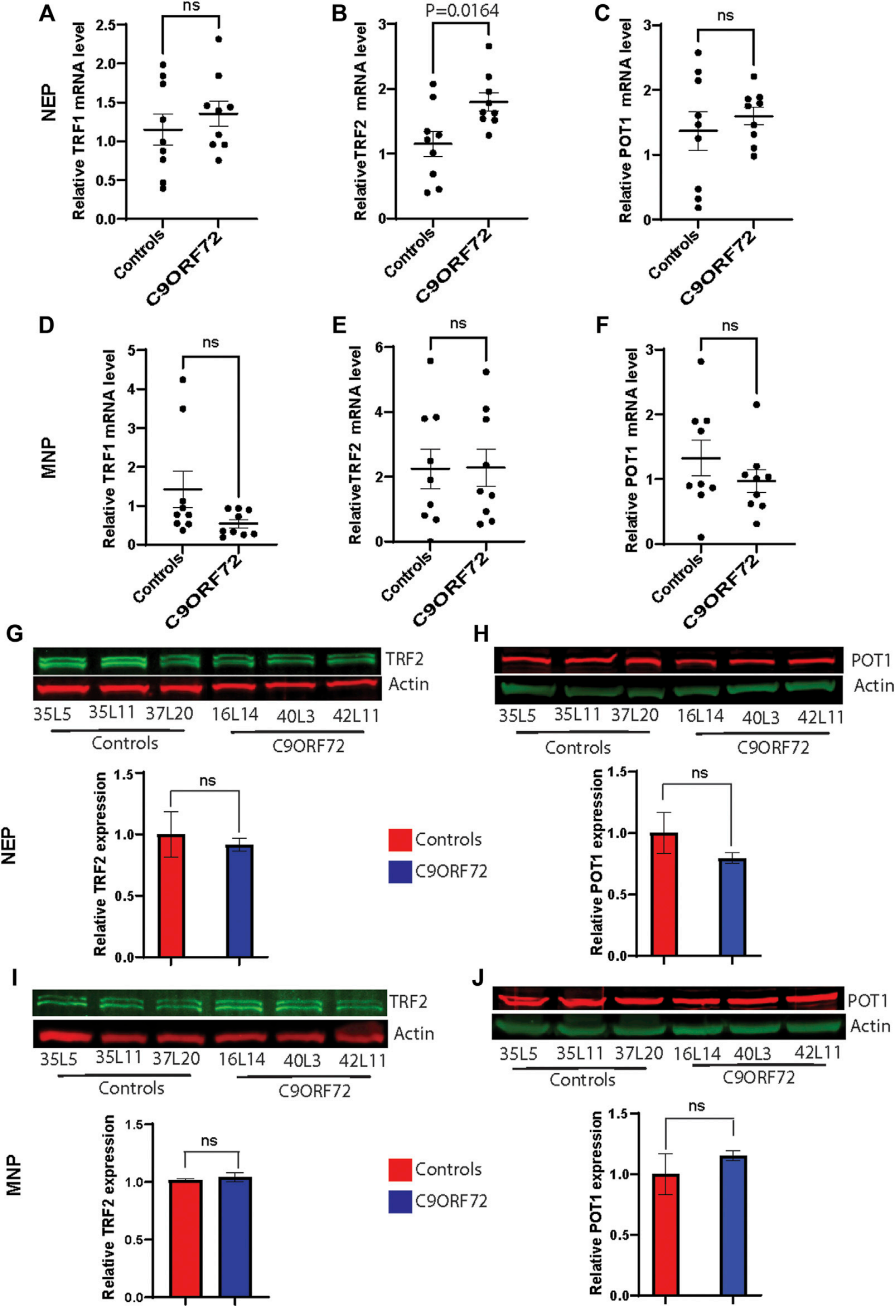
Analysis of the expression of telomere maintenance genes during the differentiation of iPSCs to MNs from controls and *C9ORF72* repeat expansion carriers. qPCR analysis to compare expression levels **(A)** TRF1, **(B)** TRF2 and **(C)** POT1 transcripts in NEP and levels of **(D)** TRF1, **(E)** TRF2 and **(F)** POT1 in MNP from 3 control subjects and 3 *C9ORF72* carriers, from 3 independent differentiations. Western blot analyses to compare protein levels of **(G)** TRF2 and **(H)** POT1 in NEP and protein levels of **(I)** TRF2 and **(J)** POT1 in MNP, from 3 controls and 3 *C9ORF72* iPSC lines, Two-tailed *t*-test with Welch’s correction was applied. ns, not significant.

To determine whether there were differences in telomere length during differentiation of iPSCs into MNs (Figure 1A), we used iPSC lines from 3 control subjects and 3 *C9ORF72* repeat expansion carriers. These lines were fully-characterized and published previously (Zhang et al., 2013; Freibaum et al., 2015; Lopez-Gonzalez et al., 2016). When we analyzed telomere length in NEP, we found no significant differences between controls and *C9ORF72* carriers (Figure 1B), on the other hand we found a significant increase in telomere length in MNP from C9ORF72 as compared with controls (Figure 1C *p* = 0.01). Then, we analyzed the relative mRNA expression of the telomere maintenance genes TRF1, TRF2, and POT1 at different stages of the MN differentiation protocol. When we analyzed NEP, we found no significant differences in TRF1 or POT1 mRNA levels. However, TRF2 mRNA levels were significantly higher in NEP from *C9ORF72* carriers (Figures 2A–C; *p* = 0.0164). For MNP, there were no significant differences between controls and *C9ORF72* carriers (Figures 2D–F). In addition we quantified TRF2 and POT1 protein levels by western blot and we did not find a significant difference between controls and *C9ORF72* carriers in the NEP stage (Figures 2G,H) or the MNP stage (Figures 2I,J). These data suggest that *C9ORF72* repeat expansion induces differential TRF2 mRNA expression, in NEP but not at the protein level, and a slight but significant difference in telomere length in MNP.

### Age-dependent Telomere Attrition and Shelterin Complex Dysregulation in *C9ORF72*-Derived MN

**FIGURE 3:**
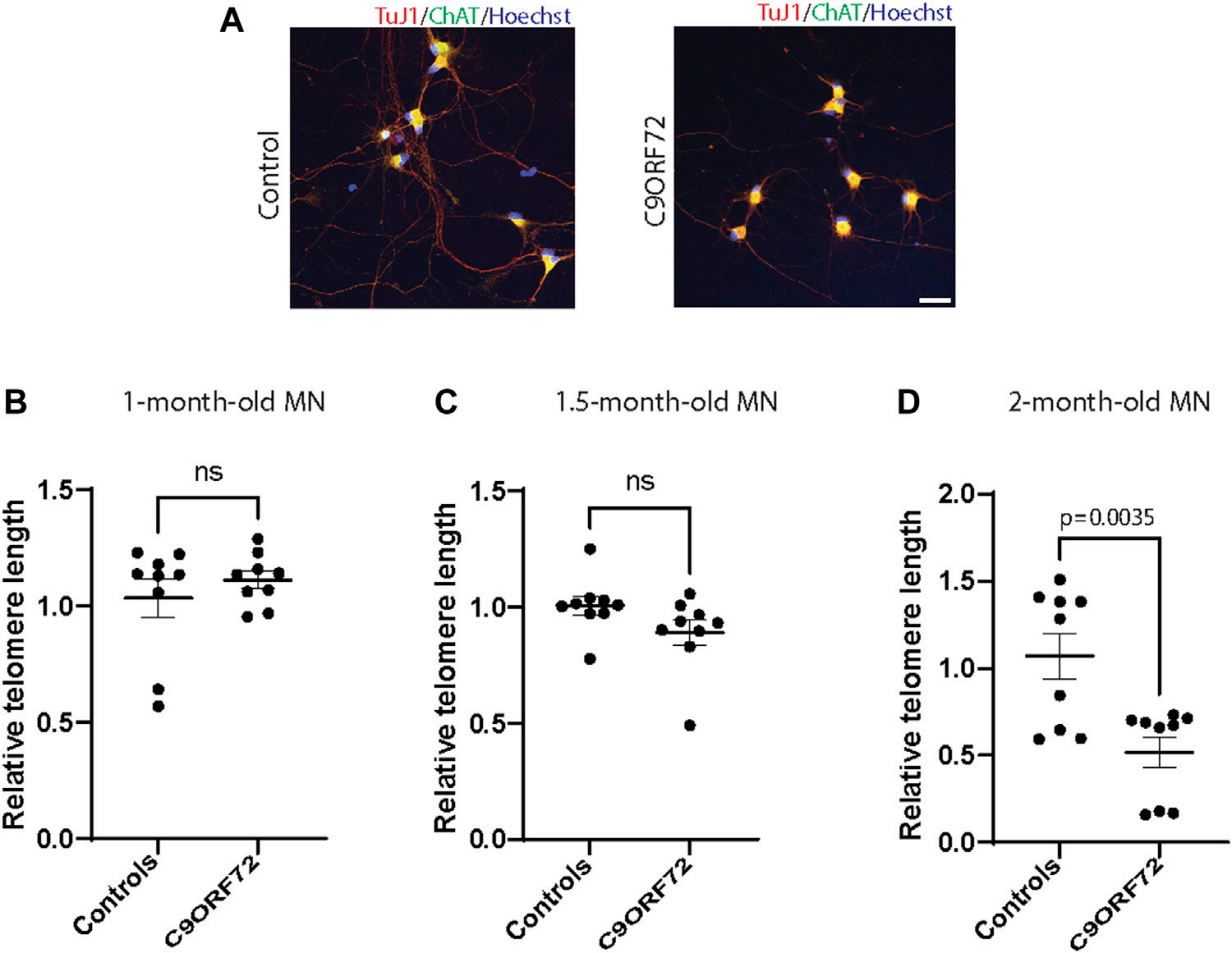
Age-dependent telomere attrition in MNs from *C9ORF72* repeat expansion carriers. **(A)** Representative images from control and *C9ORF72* iPCS-derived MN cultures. qPCR analyses of telomere length quantification in postmitotic MNs at **(B)** 1, **(C)** 1.5, and **(D)** 2 months. A one-way ANOVA was applied to compare MNs at 1 month, 1.5 months, and 2 months, from 3 controls and 3 *C9ORF72* iPSC lines. ns, not significant. Scale bar = 20 µm.

**FIGURE 4:**
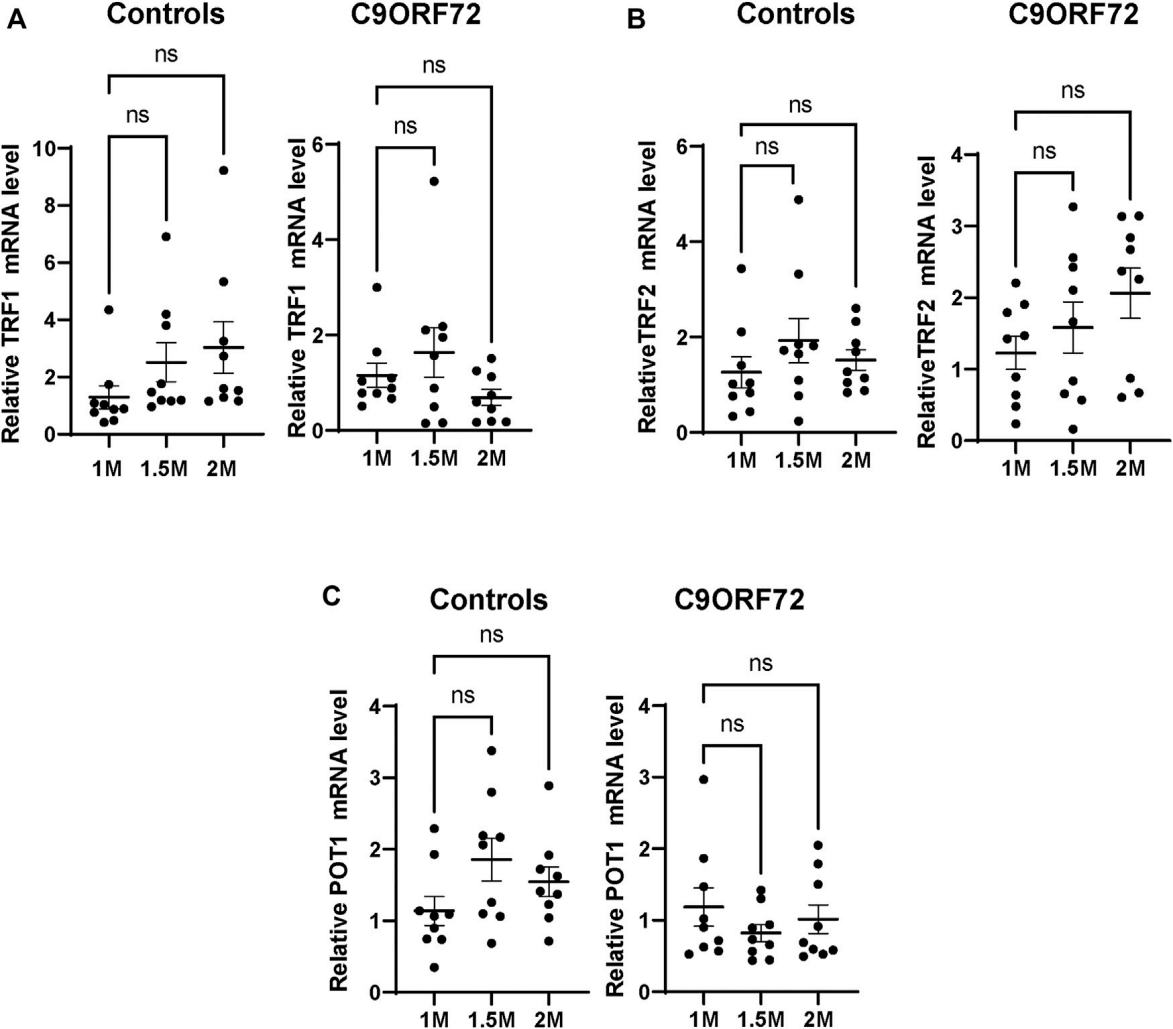
mRNA expression of telomere maintenance genes in MNs from *C9ORF72* repeat expansion carriers. qPCR analysis of the relative levels of **(A)** TRF1, **(B)** TRF2 and **(C)** POT1, mRNA. A one-way ANOVA was applied to compare iPSC-derived MNs at 1 month, 1.5 months, and 2 months from 3 control subjects or 3 *C9ORF72* carriers from 3 independent differentiation experiments. ns, not significant.

**FIGURE 5:**
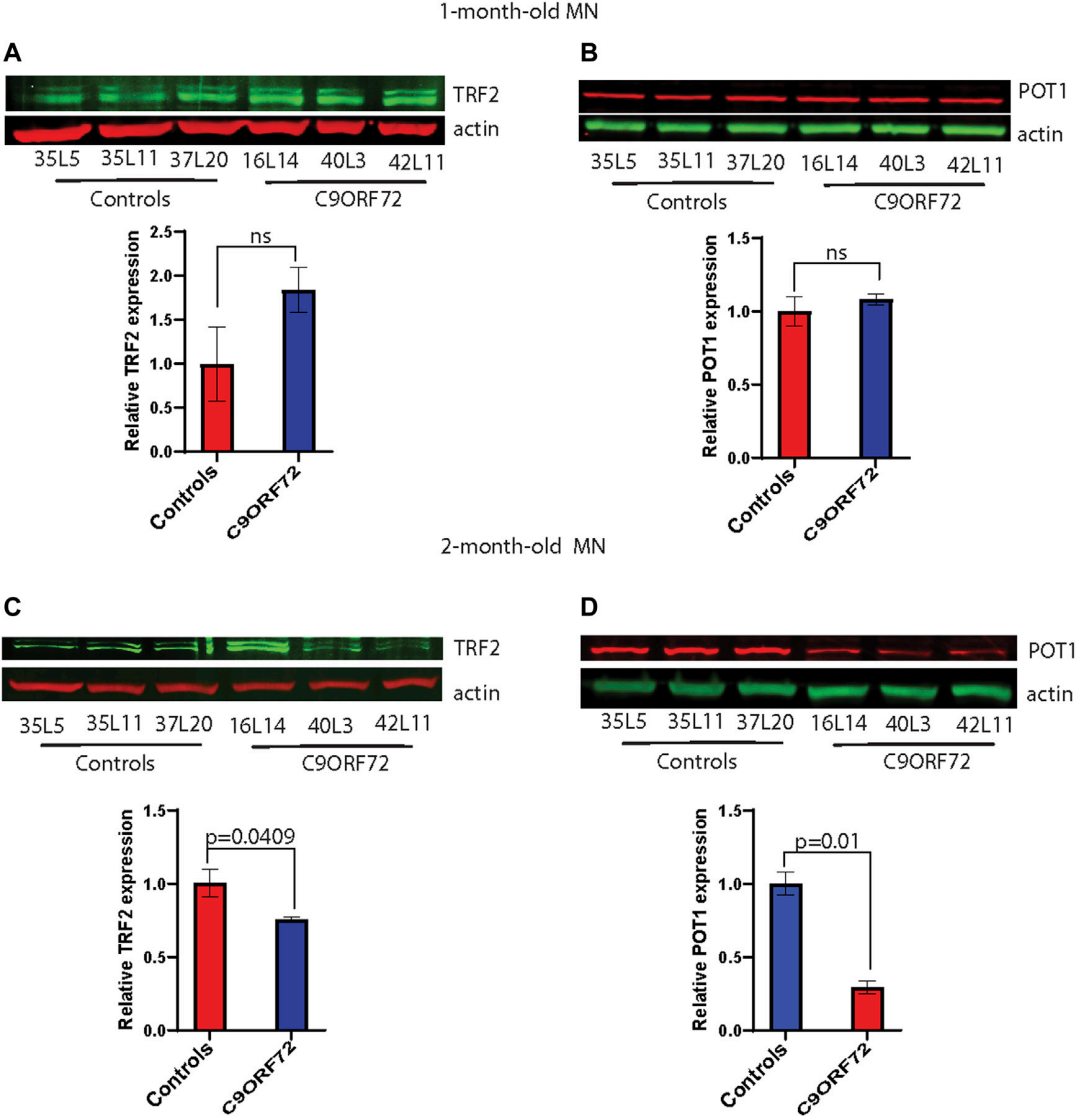
Decreased protein levels of TRF2 and POT1 in 2 month-old-motor neurons. Western blot analyses of **(A)** TRF2, **(B)** POT1 in 1-month-old-motor neurons and **(C)** TRF2, **(D)** POT1 in 2 month-old-motor neurons from 3 controls and 3 *C9ORF72* carriers. Two-tailed *t*-test with Welch’s correction was applied to western blot data.

Next, we analyzed telomere length in post-mitotic MNs differentiated from iPSCs (Figure 3A). There were no significant differences in telomere length between controls and *C9ORF72* in 1-month and 1.5 month-old MNs (Figures 3B,C). However, in 2-month-old MNs, there was a decrease in telomeric length (Figure 3D; *p* = 0.0035), which is consistent with the age-dependent increase in DNA that we and others have reported previously (Lopez-Gonzalez et al., 2016; Farg et al., 2017). To test whether the telomere attrition observed in the *C9ORF72* MNs was associated with a dysregulation in the levels of shelterin complex members, we analyzed the mRNA levels of TFR1, TRF2, and POT1 in iPSC-derived MNs from control subjects and *C9ORF72* carriers at different time points: 1 month, 1.5 months, and 2 months. TRF1, TRF2, and POT1 mRNA expression were not significantly different between *C9ORF72* and control neurons at any of the time points analyzed (Figures 4A–C). On the other hand, when we analyzed TRF2 and POT1 protein levels, we found no difference between controls and *C9ORF72* carriers in 1-month-old MNs (Figures 5A,B), when we analyzed 2-month-old, we found a decrease in TRF2 and POT1 levels in MNs from *C9ORF72* carriers as compared to controls (Figures 5C,D), indicating that the G4C2 repeat expansion induced a dysregulation in the expression of key shelterin complex members.

### Telomerase Activity and Telomerase Reverse Transcriptase mRNA Levels Motor Neurons

**FIGURE 6:**
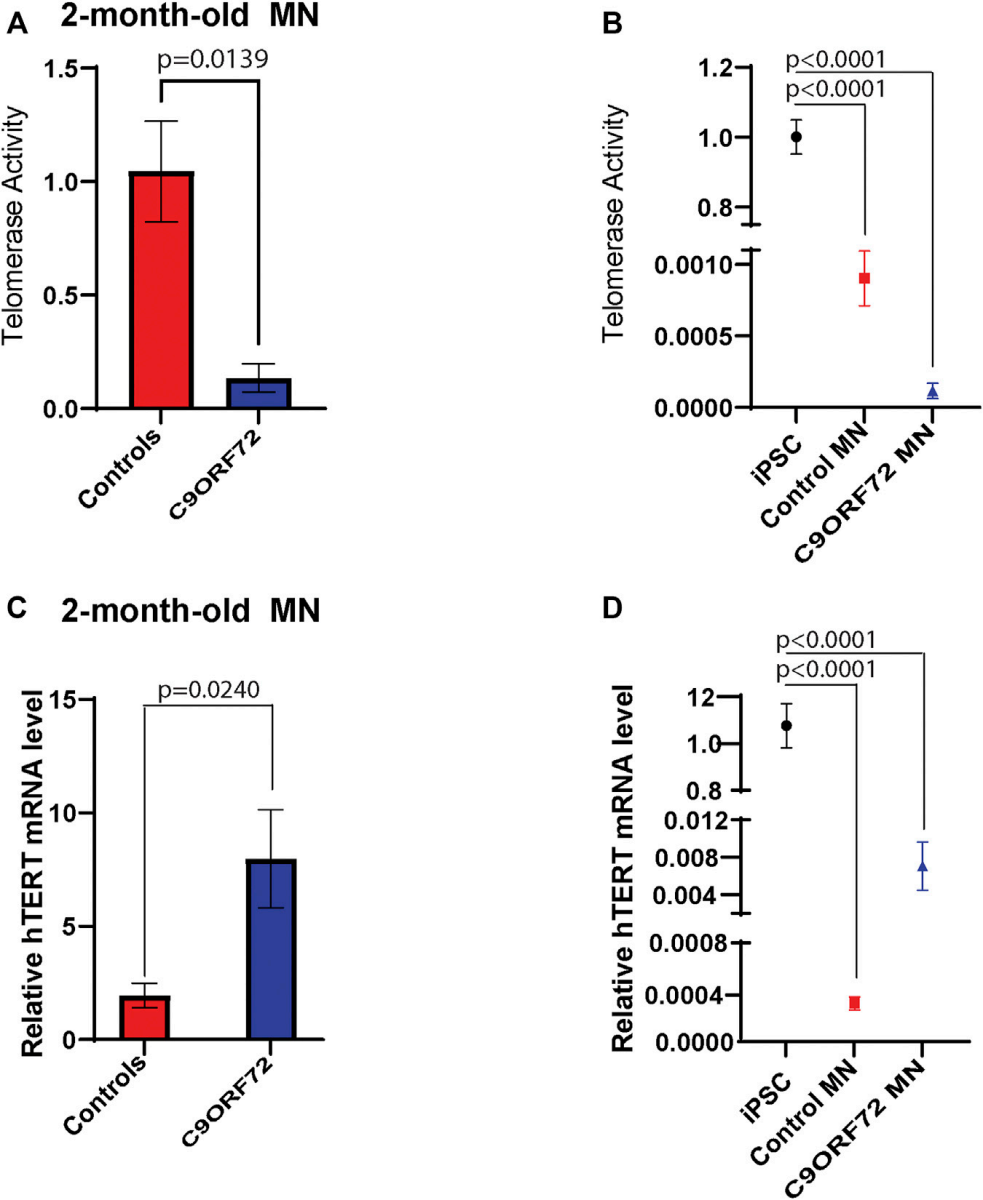
Telomerase activity and hTERT mRNA levels in 2 month-old-motor neurons. qPCR analyses of relative levels of telomerase activity in **(A)** 2 month-old iPSC-derived MNs from 3 control and 3 *C9ORF72* carriers, **(B)** relative levels of telomerase activity in iPSC lines and 2 month-old iPSC-derived MNs from 3 control and 3 *C9ORF72* carriers. qPCR analysis of the relative levels of hTERT in **(C)** 2-month-old iPSC-derived MNs from 3 control subjects or 3 *C9ORF72* carriers, **(D)** relative levels of hTERT in iPSC lines and 2 month-old iPSC-derived MNs from 3 control and 3 *C9ORF72* carriers. Two-tailed *t*-test with Welch’s correction to compare between controls and *C9ORF72* MNs and a one-way ANOVA to compare iPSC to iPSC-derived MNs cultures.

During differentiation, telomerase reverse transcriptase (TERT) levels and telomerase activity are decreased as compared to stem or cancer cells that are actively dividing. In post-mitotic cells, TERT and telomerase levels are low or undetectable. Here we analyzed telomerase activity and hTERT mRNA levels in control and *C9ORF72* iPSC-derived MNs. We found a significant decrease in telomerase activity in 2-month-old *C9ORF72* MNs as compared to controls (Figure 6A; *p* = 0.0139), It is important to note that telomerase levels in MNs were significantly lower as compared to iPSCs (Figure 6B; *p* < 0.0001). When we analyzed hTERT mRNA levels 2-month-old MNs, we found significantly higher hTERT mRNA levels in *C9ORF72* MN as compared to controls (Figure 6C; *p* = 0.0240). Similar to telomerase activity, we found lower levels of hTERT mRNA in MNs as compared to iPSCs (Figure 6D *p* < 0.0001).

## Discussion

The main goal of this study was to analyze telomere length and shelterin complex expression in iPSC-derived motor neurons from *C9ORF72* expansion carriers. There is an abundance of evidence that links telomere attrition to neurodegenerative disease (Honig et al., 2006; Scheffold et al., 2016; Guo and Yu, 2019; Nudelman et al., 2019). However, most of these studies have been performed using blood cells or postmortem brain samples (Lukens et al., 2009; Guan et al., 2015). To our knowledge, this is the first study that analyzed telomere length in patient-derived iPSC-derived neurons from *C9ORF72* repeat expansion carriers. In this study, we benefited from a well-characterized differentiation method that produces NEP, MNP, and highly pure MN populations. First, we analyzed telomere length during motor neuron differentiation and we found no differences in telomere length in NEP between controls and *C9ORF72* carriers. Interestingly, when we analyzed MNP, which is the next differentiation stage, we found an increase in telomere length in *C9ORF72* carriers as compared to controls. Subsequently, we analyzed the expression of shelterin complex genes and we only found a significant increase in TRF2 mRNA levels on NEP from C9ORF72 carriers. TRF1 and POT1 levels remained unchanged in NEP. There were no differences in TRF1, TRF2 and POT1 mRNA levels at MNP stage. When we analyzed protein levels of TRF2 and POT1, we did not find differences at either the NEP or MNP stage, suggesting that the *C9ORF72* repeat expansion induces TRF2 mRNA differential expression but this difference is not reflected at the protein levels, since protein levels remain the same at NEP and MNP in controls and *C9ORF72* carriers.

When we analyzed post-mitotic motor neurons at different time points, we found a trend towards a decrease in telomere length in *C9ORF72* neurons in 1-month-old and 1.5-month-old MNs when compared to controls. By two months, we saw a significant decrease in telomere length in *C9ORF72* MNs. Telomere length has been evaluated in FTD patients with mixed results due to the fact that different sets of samples and subjects with different forms of dementia have been analyzed. For instance, a study that analyzed blood leukocytes from 53 patients with FTD found that telomere length was increased (Kim et al., 2021). However, that study analyzed patients with different dementia variants, including FTD with behavioral variant, semantic variant primary progressive aphasia, nonfluent/agrammatic, and only 3 patients with ALS/FTD. On the other hand, a study that analyzed 70 dementia patients found shorter telomeres as compared with controls (Kota et al., 2015). For this reason, we aimed to study telomere length in a disease-relevant and well-characterized neuronal population.

The concerted action of shelterin complex proteins insulates telomeres and avoids DDR reading the linear portion of the chromosome ends as DNA breaks. Our analysis on the expression of shelterin complex members showed no differences in TRF1, TRF2, or POT1 mRNA levels in iPSC-derived MNs at any of the time points analyzed or between control and *C9ORF72* conditions. Interestingly, when we analyzed protein levels, we found decreases in TRF2 and POT1 levels in two-month-old MNs. There is evidence of a relationship between shelterin complex dysregulation and telomere shortening. TRF2 levels were found to be decreased in patients with AD (Wu et al., 2019). In addition, treatment with agents that induce ROS reduces levels of POT1 in HK-2 cells (Chuenwisad et al., 2021). Moreover, the decreases in TRF2 and POT1 levels cause unprotected chromosomes to trigger the DDR. We and others have found an increase in the DDR in *C9ORF72* neurons, which could be a result of telomere attrition.

During differentiation, TERT levels and telomerase activity are decreased as compared to stem cells that are actively dividing cells. In post-mitotic cells, TERT levels and telomerase activity are low or undetectable (Greenberg et al., 1998). In this study, we analyzed telomerase activity and hTERT mRNA levels in control and C9 iPSC-derived MNs, and we found a significant increase in hTERT mRNA levels in *C9ORF72* as compared to controls. It has been reported previously that under metabolic or oxidative stress conditions, TERT levels are increased in neurons (Kang et al., 2004) and astrocytes (Baek et al., 2004). Interestingly, oxidative stress induces TERT protein shuttling from the nucleus to mitochondria and is able to decrease ROS (Ahmed et al., 2008). On the other hand, we found a decrease in telomerase activity in 2-month-old *C9ORF72* MNs as compared to controls. It is worth noting that hTERT levels and telomerase activity are significantly lower in MNs as compared to iPSCs, with decreases of 100 and 1000 fold, respectively. For this reason, it will be interesting to explore the non-canonical functions of hTERT in neurons.

It has been demonstrated that the telomere sequence (TTAGGG)n can form G-cuadruplexes (G4). Interestingly, telomere G4 structure formation has been associated with genome instability since it can create mutations and recombination events in cancer cells (Kosiol et al., 2021). The DNA and RNA sequences from the G4C2 repeat in the non-coding region of *C9ORF72* can form G4 structures (Fratta et al., 2012), suggesting that G4 structure may also be a contributor to genomic instability in *C9ORF72* neurons. Moreover, G4 structures have been identified in many diseases, including progressive myoclonus epilepsy type 1, spinocerebellar ataxia, and other neurodegenerative diseases (Simone et al., 2015). For this reason, it will be interesting to therapeutically target this structure since there are over a thousand small-molecule ligands that can bind to G4 structures (Campbell et al., 2008), and may be valuable as future therapies.

Most of our knowledge on telomere biology comes from studies on dividing cells. However, several studies have shown telomere shortening in non-dividing cells like neurons (Lukens et al., 2009) and muscle cells (Chang et al., 2018). The consequences of telomere attrition in post-mitotic cells is still poorly understood, and it will be of interest to gain more insight into the role of telomere shortening in non-dividing cells like neurons, since this will help to illuminate the causes of neurodegeneration in ALS/FTD and other neurodegenerative diseases.

## Significance Statement

Amyotrophic lateral sclerosis (ALS) and frontotemporal dementia (FTD) are two fatal neurodegenerative diseases that unfortunately have no cure. Therefore, gaining insight into the mechanisms that drive degeneration in ALS and FTD will provide important tools to target and develop therapeutic interventions. We have previously found an increase in DNA damage and the generation of reactive oxygen species (ROS) in neurons reprogramed from patients with the *C9ORF72* mutation, which is the most common form of familial ALS and FTD. There is evidence of telomere degradation in neurodegenerative diseases, and it is well-established that telomere erosion can cause cell death or senescence. However, the evidence is inconclusive in neurodegenerative disorders, since the studies that evaluated telomere length have been performed in postmortem tissues or blood cells. Here, we analyzed telomere length in patient-derived motor neurons from *C9ORF72* carriers, a disease-relevant neuronal type. We found reduced telomere length in mature neurons and dysregulation of the expression of telomere-associated proteins on *C9ORF72* neurons as compared to control subjects.

## Data Availability

The raw data supporting the conclusions of this article will be made available by the authors, without undue reservation.
